# Comprehensive Investigation of Grade-Specific Aroma Signatures in Nongxiangxing Baijiu Using Flavoromics Approaches

**DOI:** 10.3390/foods14213781

**Published:** 2025-11-04

**Authors:** Yangyang Sun, Heyun Zhang, Huan Zhang, Jihong Huang, Liping Du, Juan Wang

**Affiliations:** 1 Collaborative Innovation Center of Functional Food by Green Manufacturing, The Food and Pharmacy College, Xuchang University, Xuchang 461000, China; 2 State Key Laboratory of Food Nutrition and Safety, Key Laboratory of Industrial Fermentation Microbiology Ministry of Education, Tianjin Key Laboratory of Industrial Microbiology, College of Biotechnology, Tianjin University of Science and Technology, Tianjin 300457, China; heyzhang09@163.com (H.Z.); zhanghuan90@tust.edu.cn (H.Z.); 3 Liquor Making Bio-Technology and Application of Key Laboratory of Sichuan Province, Sichuan University of Science and Engineering, Yibin 644000, China; 4 School of Food Science and Engineering, South China University of Technology, Guangzhou 510641, China

**Keywords:** nongxiangxing baijiu, GC-MS, electronic nose, aroma recombination and omission, key aroma compound

## Abstract

At present, the classification of nongxiangxing baijiu (NXB) predominantly relies on the subjective expertise of distillers and tasters, in the absence of scientifically standardized criteria. This reliance poses challenges for achieving precise classification. Consequently, there is an urgent need to employ flavoromics technology to identify the key characteristic aroma compounds that distinguish different grades of NXB. This study uses sensory evaluation, an electronic nose, and three-dimensional fluorescence spectroscopy to determine the significant differences in aroma profiles among different grades of NXB. Further, 112 compounds were identified by GC-MS, and quantitative analysis was conducted on 33 major volatile compounds. Using odor activity value (OAV) analysis, it found 21, 23, and 26 key aroma compounds (OAV ≥ 1) in Grade 2 (G2), Grade 1 (G1), and prime-grade (GP), respectively. Aroma recombination successfully simulated the aroma distribution of different grades of NXB, and omission experiments identified 11 key aroma compounds as the foundation for NXB. Additionally, each grade exhibited a unique profile of key compounds. G2 was characterized by the presence of octanoic acid, which imparts sweaty and cheesy aromas. G1 contained benzoic acid ethyl ester, phenethyl acetate, pentanoic acid, 2-methyl-1-propanol, and furfural, contributing to sweet, floral, sweaty/cheesy, alcoholic, and roasted aromas. GP demonstrated higher concentrations of aroma compounds compared to G1 and G2, with distinctive compounds such as decanoic acid ethyl ester, dodecanoic acid ethyl ester, 3-methyl-1-butanol, and 1-hexanol, which are associated with sweaty/cheesy, fruity, and alcoholic aromas. This study elucidated the role of these compounds in determining the flavor profiles of various NXB grades, thereby providing a theoretical foundation for the quality grading of NXB.

## 1. Introduction

As one of the world’s six major distilled spirits, Chinese baijiu has undergone over 2000 years of development, bearing rich cultural connotations, and is regarded as the “national drink” [1]. Based on distinct brewing conditions and flavor characteristics, Chinese baijiu is classified into 12 aroma types, among which nongxiangxing, jiangxiangxing, and qingxiangxing baijiu are the oldest and most popular. Qingxiangxing baijiu has a typical style of “pure aroma and refreshing taste”. Jiangxiangxing baijiu has a typical style of “refined aroma, rich taste and enduring aftertaste”. Nongxiangxing baijiu (NXB), with its typical style of “rich cellar aroma, smooth and sweet taste, well-coordinated flavors, and a clean, long aftertaste”, dominates the market compared with qingxiangxing and jiangxiangxing baijiu. The production of NXB primarily uses sorghum as the raw material, supplemented with recycled fermented grains, and employs medium-temperature Daqu as the saccharification and fermentation agent. These components are mixed and placed in a mud pit, a unique fermentation vessel, for fermentation. This step is the most crucial process in brewing NXB, as it imparts the distinctive cellar aroma characteristic of the baijiu. The fermented grains are then distilled to obtain base liquor, which is aged to produce the finished product. The typical alcohol content of NXB is 52% vol. This degree is considered to be the “golden degree” of NXB, which makes the compound aroma of baijiu best displayed. As a typical representative of traditional Chinese distilled liquor, its quality characteristics are closely related to its aromatic components. Due to the diverse raw materials and complex production processes, it has a rich composition. These trace components, mainly esters, acids, alcohols, aldehydes, sulfur-containing, nitrogen-containing, and heterocyclic compounds, vary in type and content. So far, 861 trace components have been identified in NXB [2].

In recent years, through flavoromics technology, research on NXB has made great progress. Mu et al. [3] used Headspace Solid-Phase Microextraction (HS-SPME) combined with Comprehensive Two-Dimensional Gas Chromatography-Time-of-Flight Mass Spectrometry (GC×GC-TOF MS) to identify 168 volatile compounds in fresh nongxiangxing baijiu. Among them, 29 compounds, especially ethyl hexanoate and ethyl lactate, with high OAVs, were further identified as aroma-active compounds. Zhao et al. [4] identified 60 aroma compounds in NXB (Gujinggong baijiu), and determined ethyl hexanoate, ethyl octanoate, ethyl pentanoate, butyric acid, and γ-decalactone as key aroma compounds. He et al. [5] combined GC×GC-TOF MS with sensory description analysis, revealing region-specific characteristics of NXB. There were 58 aroma compounds that could mark the significant differences between samples from Sichuan and Jianghuai areas. Hong et al. [6] identified 78 aroma compounds in NXB from northern China. He et al. [7] used liquid–liquid microextraction (LLME) combined with gas chromatography-mass spectrometry (GC-MS) and headspace gas chromatography-ion mobility spectrometry (HS-GC-IMS) to monitor volatile compound changes during NXB distillation, and found ethyl butyrate and ethyl hexanoate could distinguish head liquor samples. Li et al. [8] employed GC×GC-MS to elucidate the distribution of flavor components contributing to the aging of NXB, laying the foundation for identifying aging-related flavor markers. Most current studies on NXB focus on aroma compound identification, regional aroma differences, and changes in aroma compounds during distillation and storage. However, research on the differences in aroma components among different grades of NXB remains limited, crucial for quality evaluation, and the difference in these aroma components plays an important role in the quality of NXB.

As we all know, the liquor obtained through distillation is called base liquor. In liquor production, distilled base liquor is graded into different categories (including second-grade baijiu, first-grade baijiu, and prime grade baijiu) based on blending processes. Currently, this classification relies heavily on the experience of liquor makers and tasters, lacking scientific and uniform standards, which hinders accurate grading and the healthy development of the liquor industry. Thus, it is essential to apply flavoromics techniques to analyze different-grade NXB and determine their key characteristic compounds. This will provide a theoretical basis for the identification of grade NXB.

In this study, three grades of NXB and a base liquor as a control were analyzed. Using a multi-technique integrated flavoromics approach, we aimed to comprehensively characterize the flavor profiles of different-grade NXB. First, sensory analysis, electronic nose, and 3D fluorescence spectroscopy were used to evaluate their aroma profiles. This aimed to verify whether measurable differences exist in the overall flavor structure of different grades of Baijiu. Then, GC-MS and GC-FID were applied for qualitative and quantitative analysis of volatile compounds. Finally, key aroma compounds of different grades NXB were determined through OAV analysis, aroma recombination, and omission tests. This study will provide a theoretical basis for classification of different grades of NXB.

## 2. Materials and Methods

### 2.1. Sample Collection

Experimental samples of different grades of NXB were sourced from a specific distillery. This manufacturer is a representative manufacturer in this area. Different batches and grades of products have been tested repeatedly, and the results prove that the products of the same grade have high stability and consistency in flavor components. Therefore, a representative group of samples was selected. The samples were named as grade 2 baijiu (G2), grade 1 baijiu (G1), and prime-grade baijiu (GP), and the base liquor (B1) was selected as the control.

### 2.2. Reagents

Ethyl acetate, pentanoic acid ethyl ester, hexanoic acid ethyl ester, octanoic acid ethyl ester, benzoic acid ethyl ester, pentanoic acid, 2-methyl-1-propanol and 3-methyl-1-butanol were purchased from Meryer (Shanghai, China). Butanoic acid ethyl ester, heptanoic acid ethyl ester, 2-hydroxy-propanoic acid ethyl ester, hexanoic acid hexyl ester, benzeneacetic acid ethyl ester, acetic acid 2-phenylethyl ester, benzenepropanoic acid ethyl ester, dodecanoic acid ethyl ester, linoleic acid ethyl ester, tetradecanoic acid ethyl ester, hexadecanoic acid ethyl ester, ethyl 9-hexadecenoate, acetic acid, propanoic acid, hexanoic acid, 1-propanol, 1-pentanol, 1-hexanol, acetaldehyde, and furfural were purchased from Macklin (Shanghai, China). Decanoic acid ethyl ester was purchased from Sigma-Aldrich (Shanghai, China). Butanoic acid, heptanoic acid, octanoic acid, phenylethyl alcohol, sodium chloride, and ethanol were purchased from Aladdin (Shanghai, China). All the above chemicals are chromatographically pure (purity greater than 98%).

### 2.3. Sensory Evaluation

Sensory analysis on NXB aroma was conducted according to the method of Xiao et al. [9]. Twelve panelists (4 males and 8 females) with experience in olfactory experiments and quantitative descriptive analysis were recruited from our laboratory. All participants signed informed consent to participate. The experiment has passed ethical review; proof was provided in Figure S1. Each panelist has received no less than 90 h of good training over 3 months, which lasts for one semester. Finally, ten trained panelists, who consisted of six females and four males from 23 to 29 years old, were screened out according to their sensitive olfaction and the correct description of the aromas. After systematic training and practice, characteristic aromas of baijiu samples were discussed, and eight scents were finally determined, including “fermented”, “fruity”, “alcoholic”, “sour aromatic”, “sweaty/cheesy”, “grainy”, “sweet aromatic”, and “floral” [10,11]. “Fermented” reference standard is hexanoic acid ethyl ester; “Fruity” reference standard is butanoic acid ethyl ester; “Alcoholic” reference standard is ethanol; “Sour aromatic” reference standard is acetic acid; “Sweaty/cheesy” reference standards are butyric acid and hexanoic acid; “Grainy” reference standard is steamed sorghum; “Sweet aromatic” reference standard is γ-nonalactone; “Floral” reference standard is phenethyl alcohol. Then, four kinds of baijiu samples (30 mL each) were randomly coded and transferred into standardized wine tasting glasses conforming to national specifications [12]. Trained panelists then performed olfactory assessments to determine aroma intensity scores. During the whole sensory analysis, samples were evaluated in standard sensory booths, with white lighting, controlled airflow, and a room temperature of 20 ± 1 °C [13]. A standardized 6-point intensity scale (0–5) was employed for odor descriptor quantification, where 0 points (not perceptible) indicated that the aroma attribute cannot be detected in the sample; 1 point indicated that the intensity is just barely perceptible; 2 points (slight) indicated that the intensity is weak but clearly present; 3 points (moderate) indicated that it possesses a distinct intensity; 4 points (strong) indicated that the intensity is high, representing a dominant characteristic of the sample; and 5 points (extremely strong) indicated the highest perceived intensity for that aroma attribute [14]. To ensure methodological rigor, this evaluation protocol was systematically replicated in triplicate for each sample.

### 2.4. Electronic Nose Analysis

Electronic nose analysis was conducted using a modified protocol adapted from Qiu et al. [15]. 25 μL of sample was mixed with 975 μL of ultrapure water in a 20 mL headspace vial followed by immediate sealing, with 250 μL injected at 100 μL/s through a 200 °C injection port. The system employed dual capillary columns (MXT-5 and MXT-1701 (RESTEK, Bellefonte, PA, USA), both 2 m × 0.18 mm) coupled with hydrogen carrier gas in constant flow mode (1 mL/min), featuring operational parameters including a 50 °C trap temperature, 60 kPa column head pressure, and 30 mL/min split flow. The temperature program was initiated at 50 °C (2 s hold), ramped at 1 °C/s to 80 °C, then accelerated to 250 °C at 23 °C/min (15 s final hold), while dual flame ionization detectors maintained at 260 °C ensured compound detection. Triplicate analyses were systematically performed for all samples to ensure analytical reliability.

### 2.5. Three-Dimensional Fluorescence Spectroscopy Analysis

Fluorescence spectral analysis was performed using a Hitachi FL-7100 spectrophotometer (Hitachi High-Tech Corporation, Tokyo, Japan) under optimized instrumental conditions. For each measurement, 1 mL of sample solution was transferred into a standard quartz cuvette (10 mm path length). Parameters were set as follows: a xenon lamp was used as the light source; excitation wavelength (EXWL) was 200–600 nm, and step length was 5 nm; emission wavelength (EMWL) was 200–600 nm, and step length was 5 nm; slit width was 5 nm; scan speed was 30,000 nm/min; response time was Auto mode; and PMT voltage was 500 V.

### 2.6. GC-MS Analysis of Volatile Compounds

#### 2.6.1. Headspace Solid-Phase Microextraction (HS-SPME)

The methods referred to the procedures by Sun et al. [16]. The sample was diluted to achieve an ethanol content of 10% (*v*/*v*) (8 mL), and sodium chloride (3 g) was added to headspace vial. An internal standard solution (407.5 mg/L of 2-octanol and 418.8 mg/L of pentyl acetate) was added to sample in a volume of 20 μL, then the headspace vial was tightly capped with a PTFE-silicon septum. The 50/30 µm divinylbenzene/carboxen/polydimethylsiloxane (DVB/CAR/PDMS) fiber purchased from Supelco (Bellefonte, PA, USA), was inserted into the headspace of the sample vial. The sample vial was equilibrated at 60 °C for 10 min and extracted for 45 min. In particular, the sample needed to be shaken at 400 rpm. It is worth noting that this extraction method is the best method obtained by optimizing the extraction conditions through the early methodological research in our laboratory. Under these conditions, the target compound can be extracted most comprehensively, to obtain the analytical data with a high signal-to-noise ratio and strong representation. All volatile compounds absorbed on SPME fiber were desorbed at the GC injector at 250 °C for 5 min. Each analysis was repeated three times.

#### 2.6.2. GC-MS

GC-MS analysis was performed on a GC system (Agilent 7890A, Agilent Technologies Inc., Santa Clara, CA, USA) equipped with a mass spectrometer (Agilent 5975C, Agilent Technologies Inc., Santa Clara, CA, USA). An Agilent CP-WAX 57CB capillary column (60 m × 250 μm × 0.40 μm film thickness) (Agilent Technologies, Santa Clara, CA, USA) was employed for separating volatile compounds. Helium (purity > 99.999%) was used as carrier gas with a constant flow rate of 1 mL/min. The GC oven temperature was set at 40 °C, ramped at a rate of 10 °C/min to 50 °C and held for 5.5 min, then increased at a rate of 3 °C/min to 80 °C and kept for 8 min, lastly, it increased to 200 °C at a rate of 4 °C/min and kept for 13 min. Injector temperature was set at 250 °C with a spitless model. Mass spectra were recorded in full scan mode, and the range of the scan was set at *m*/*z* 35–350 atomic mass units (amu).

Retention indices (RI) of detected volatile components were calculated from the retention time of n-alkanes (C5–C36). Volatile compounds were identified based on retention indices (RI) and mass spectra matching in the standard NIST 20 library.

### 2.7. Quantitative Analysis of Volatile Compounds by GC-FID

Baijiu samples were filtered through a 0.22 μm organic-phase microporous membrane. An internal standard (418.8 mg/L of pentyl acetate) was added, and the mixture was homogenized before injection. The samples were separated using a CP-WAX 57CB column (50 m × 250 μm × 0.2 μm). The injector temperature was 250 °C, and the detector temperature was 260 °C. High-purity nitrogen was used as carrier gas with a constant flow rate of 1 mL/min. The GC oven temperature was set at 35 °C and held for 1 min, then increased to 70 °C at a rate of 3 °C/min, and finally increased to 190 °C at a rate of 3.5 °C/min and held for 22 min.

For each target compound to be quantified, we used its own high-purity standard. Accurately weigh appropriate amounts of each standard compound, dissolve them with chromatographic absolute ethanol, and transfer to a volumetric flask (10 mL) to prepare stock solutions. Each standard was precisely diluted to prepare a series of standard solutions at six different concentration levels. The internal standard was added to each of these solutions. This series of standard solutions was analyzed using the exact same GC-FID conditions as the actual samples. Using the ratio of the standard compound peak area to the internal standard peak area as the x-axis, and the ratio of the standard compound concentration to the internal standard concentration as the y-axis, a standard curve of standard compounds was established. The correlation coefficient (R^2^) for each calibration curve was required to be greater than 0.99 to ensure the reliability of the quantitative model. For each concentration level, we performed three replicate experiments to assess the precision of the method. After establishing the calibration curves, the Baijiu samples were analyzed to obtain the peak areas of the target compounds. Subsequently, the “target compound peak area, internal standard peak area, and internal standard concentration” were input into the corresponding calibration curve to precisely calculate the concentration of that compound in the sample.

### 2.8. Calculation of Odor Activity Values (OAVs)

The OAV is the ratio of an aroma compound’s concentration to its odor threshold. Compounds with OAV ≥ 1 are considered to significantly impact the overall aroma of baijiu. In this study, OAV calculations were systematically performed by the follwoing: (1) utilizing qualitative and quantitative analytical data of volatile compounds obtained through GC-MS and GC-FID and (2) compiling odor threshold values from ten authoritative references [1,9,14,17,18,19,20,21,22,23].

### 2.9. Aroma Recombination and Omission Experiments

For the aroma recombination experiment, a 52% (*v*/*v*) ethanol—water solution was used as the matrix. Aroma compounds with OAV ≥ 1 from the samples were added sequentially to the matrix at quantified concentrations [24]. After shaking and balancing for 10 min, sensory evaluation was conducted to compare the differences between the reconstructed model and the real baijiu sample.

In the omission experiment, an omission recombination model was created by removing one or a class of compounds from the complete recombination model. Sensory evaluation was conducted to compare the differences between the omission and complete models. Statistical significance was used to further identify key aroma compounds.

### 2.10. Statistical Analysis

All assays were conducted in triplicate, and results were presented as mean with standard deviations. Statistically significant differences were processed using one-way analysis of variance (ANOVA) in SPSS V16.0 (*p* < 0.05, Duncan’s test). Origin 2024 was used to create plots showing changes in aroma compound content and radar charts. Principal component analysis (PCA) and discriminant factor analysis (DFA) were performed using software from an electronic nose. Heatmaps, upset plots, and Venn diagrams were performed by the genescloud tools online platform (https://www.genescloud.cn (accessed on 20 May 2025)).

## 3. Results and Discussion

### 3.1. Aroma Profiles of NXB in Different Grade

#### 3.1.1. Sensory Evaluation of NXB in Different Grade

**Figure 1:**
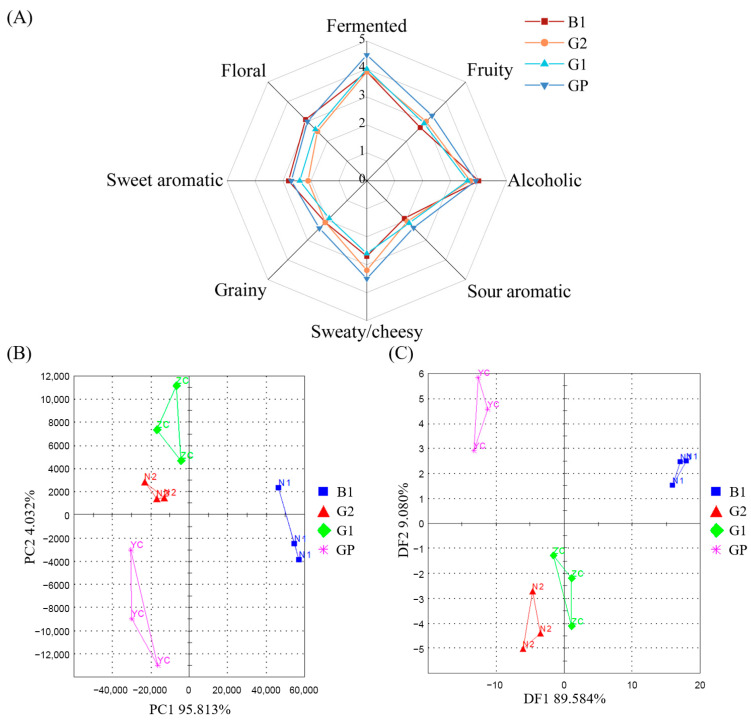
Aroma profiles of different grades of NXB (**A**) aroma radar chart; (**B**) principal component analysis (PCA); (**C**) discriminant factor analysis (DFA).

A sensory panel evaluated the aroma characteristics of NXB in different grades, with results shown in Figure 1A. There were slight differences in the sensory scores of NXB in different grades, mainly in fermented aroma and sweaty/cheesy aroma, which was consistent with previous studies [25]. These eight attributes can well explain the aroma characteristics of NXB samples in different grades. Among them, GP samples scored the highest in fermented, alcohol, sweaty/cheesy, fruity, sour aromatic, and grainy aromas, indicating the best aroma profile. G1 scored high in fermented and alcohol aromas. G2 stood out in sweaty/cheesy aroma with similar scores in grainy and sweet aromas. B1 had the highest scores in sweet, floral, and alcohol aromas but relatively low scores in other attributes. These results showed that the aroma profiles of NXB in different grades were similar in style but differed in intensity, especially in fermented and sweaty/cheesy aromas. Additionally, according to literature reports, floral, fruity, and sweet aromas have also been proved to be important attributes of NXB flavor [16].

#### 3.1.2. Electronic Nose Analysis of NXB in Different Grade

To further validate the differences in aroma profiles among different grades of NXB, PCA and DFA analyses of volatile compounds were conducted using an electronic nose. In Figure 1B, PC1 and PC2 contributed 95.81% and 4.03%, respectively, with a cumulative contribution of 99.84%, indicating that these two components captured most of the information. The four baijiu samples with different grades showed distinct, non-overlapping distributions, reflecting good within-group consistency and between-group separation. G2, G1, and GP samples clustered on the left side of the *X*-axis, with minimal differences between G2 and G1, while B1 samples were on the right side of the *X*-axis. This showed that there is a significant difference between base liquor and different grades of NXB. DFA can classify and identify more than two sample types. As shown in Figure 1C, discriminant factors DF1 and DF2 explained 89.58% and 9.08% of the variance, respectively, with a total of 98.66%, indicating good preservation of original data. The DFA also showed clear distinctions between the different grades of NXB. In conclusion, both PCA and DFA effectively discriminated between the different grades of NXB.

#### 3.1.3. Three-Dimensional Fluorescence Spectroscopy Analysis of NXB in Different Grade

**Figure 2:**
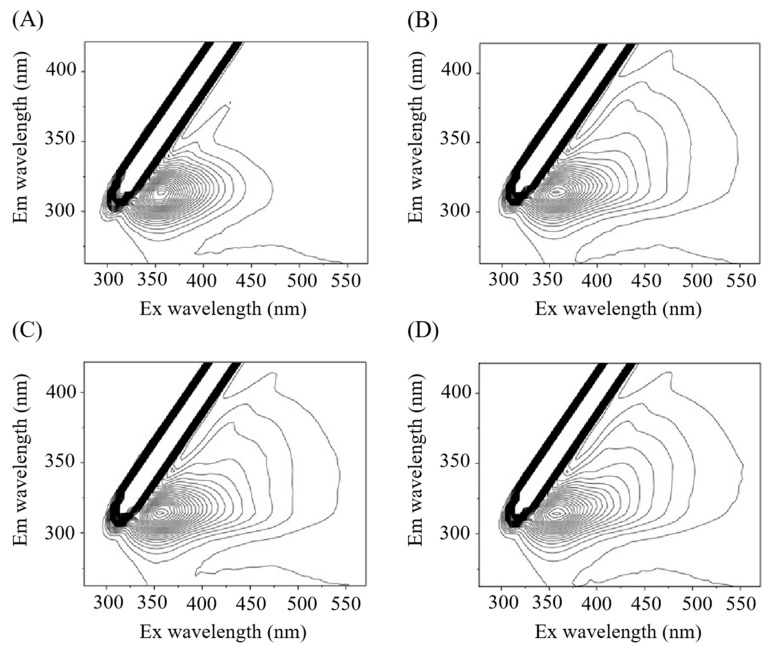
Three-dimensional fluorescence spectra of different grades of NXB (**A**) B1; (**B**) G2; (**C**) G1; (**D**) GP.

Three-dimensional fluorescence spectroscopy encompasses all spectral information of fluorescent substances. The unique peak positions and shapes in 3D spectra enable the identification of sample components [26]. This technique, known for its simplicity and efficiency, has been widely used in environmental monitoring [27], food quality testing [28], and other fields. Studies have combined 3D fluorescence spectroscopy with chemometrics to establish rapid detection methods for authenticating foods like green tea, vinegar, and wine [29,30,31,32]. Based on this, an attempt was made to distinguish NXB of different grades by using 3D fluorescence spectroscopy. The contour map was drawn in Figure 2. All four grade samples showed fluorescence peaks at 353 nm and 314 nm with an optimal excitation wavelength of 314 nm. However, compared to B1 (275–378 nm), the effective excitation wavelength ranges of different grades of NXB were broader: 275–415 nm for G2, 275–472 nm for G1, and 275–475 nm for GP. This was attributed to the complex chemical, physical, and structural information of NXB, which generated broad, overlapping fluorescence bands. Despite the complex factors affecting the aroma compounds of NXB in different grades, their unique fluorescence characteristics suggest the feasibility of using spectroscopy for rapid preliminary identification of different grades of NXB.

The combined results of sensory evaluation, electronic nose analysis, and three—dimensional fluorescence spectroscopy showed that B1 differed significantly from the graded baijiu. Among different grades of NXB, G1, G2, and GP also exhibited notable differences, with GP having the best flavor profile. This study demonstrated that an electronic nose and three-dimensional fluorescence spectroscopy can distinguish between different grades of NXB, offering a theoretical basis for its rapid grading. To further investigate the differences between samples, GC-MS was used to analyze the aroma compounds in each sample.

### 3.2. The Types of Volatile Compounds of NXB in Different Grades

HS-SPME combined with GC-MS was used to analyze the flavor components in different grades of NXB. A total of 112 compounds were identified, including 51 esters, 18 alcohols, 10 ketones, 7 acids, 3 aldehydes, 2 phenols, 1 furan compound, 4 aromatic compounds, and 16 other compounds.

**Figure 3:**
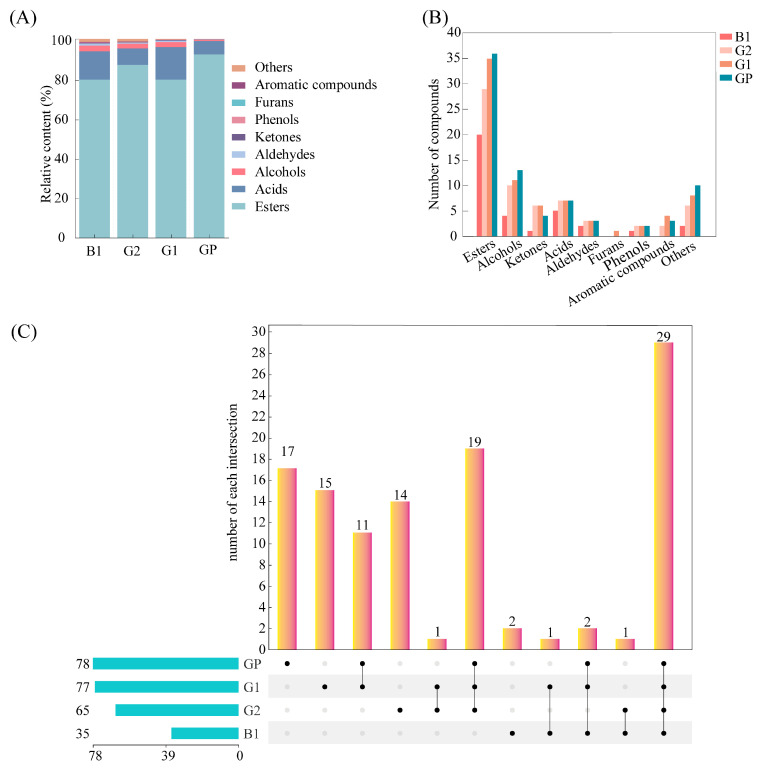
Flavor compounds in different grades of NXB (**A**) relative content; (**B**) types and quantities; (**C**) upset plot.

Esters were the most abundant compounds in NXB, accounting for 79.53–92.23% of the total flavor components (Figure 3). Among them, the relative content of hexanoic acid ethyl ester in different grades of samples was high. Hexanoic acid ethyl ester, a key compound with high content, a low odor threshold, and sweet fruity notes, significantly contributes to the main aroma characteristics of NXB. The other three major esters, including ethyl acetate, ethyl lactate, and butanoic acid ethyl ester, were also highly prevalent, together forming the “four major esters” of NXB [2].

Acids were the second most abundant compounds, making up 6.83–16.44% of the total flavor components (Figure 3). The three main acids, including acetic acid, hexanoic acid, and butyric acid, typically impart cheese and fermentation notes. The lingering taste of baijiu was closely related to some high boiling point organic acids. Combined, esters and acids accounted for 93.88–98.61% of the total flavor components, aligning with previous studies that reported esters and acids as the primary aromatic compounds in NXB [33].

Other compounds, including alcohols (0.46–2.78%), aldehydes (0.17–1.09%), ketones (0.1–0.35%), phenols (0.16–0.28%), aromatic compounds and furans (0.00–0.36%), and others (0.02–1.37%), were relatively less abundant. Alcohols were important for their alcoholic and fruity notes and served as a bridge between acidity and flavor, enhancing the aroma of esters. Alcohols mainly included 1-butanol, 2-methyl-1-propanol, 3-methyl-1-butanol, and 1-propanol. However, excessive alcohol content can lead to disharmony in aroma and taste. Aldehydes and ketones, with their grassy and fruity notes. Aldehydes mainly included acetaldehyde and furfural, which played a role in enhancing aroma and coordinating taste. Sulfur-containing compounds have cabbage- or onion-like odors, while nitrogen-containing compounds provide nutty or roasted notes. Phenols contribute phenolic and smoky flavors. These compounds not only impart their own flavors but also interact to create richer aromatic profiles, enhancing the aroma quality of NXB.

All kinds of volatile compounds were further statistically analyzed. As shown in Figure 3C, there were 29 volatile compounds in all samples, of which 16 were esters, including ethyl acetate, butanoic acid ethyl ester, pentanoic acid ethyl ester, hexanoic acid ethyl ester, propyl hexanoate, heptanoic acid, ethyl ester, ethyl lactate, butyl hexanoate, octanoic acid ethyl ester, amyl hexanoate, hexanoic acid hexyl ester, ethyl furoate, decanoic acid ethyl ester, benzeneacetic acid ethyl ester, benzenepropanoic acid ethyl ester, and hexadecanoic acid ethyl ester. GP samples had the most volatile compounds (78), including 17 unique compounds. G1 samples had 77 volatile compounds with 15 unique compounds. G2 had 65 volatile compounds with 14 unique compounds. B1 had the fewest at 35 volatile compounds with two unique compounds. Thus, B1 showed the least flavor diversity and the greatest difference from graded baijiu. Subsequent quantitative analysis of the samples was performed via GC-FID.

### 3.3. Quantitative Analysis of Volatile Compounds

Since esters, acids, alcohols, and aldehydes account for over 97% of the total compounds, quantitative analysis focused on volatile compounds with a relative content of more than 0.5% in these four categories. It is worth noting that while certain trace compounds might possess high OAVs due to very low odor thresholds and thus be key aroma compounds, the group of high-concentration compounds collectively forms the foundational flavor matrix of baijiu. The ratios and absolute differences in their concentrations are highly likely to exhibit systematic and distinguishable patterns across different quality grades. A total of 33 volatile compounds were quantified, including 18 esters, 6 alcohols, 7 acids, and 2 aldehydes, as shown in Table 1.

Esters, which were the key aroma compounds and the most abundant in NXB, had the highest content. Among them, hexanoic acid ethyl ester (558.60–1348.93 mg/L) was the most prevalent, followed by ethyl acetate (492.16–1211.82 mg/L), ethyl lactate (337.98–838.89 mg/L), and butanoic acid ethyl ester (40.94–166.47 mg/L). Other esters like decanoic acid, ethyl ester (0.05–74.44 mg/L), hexadecanoic acid ethyl ester (46.23–72.31 mg/L), and pentanoic acid ethyl ester (4.33–29.10 mg/L) were also relatively abundant. The content of dodecanoic acid ethyl ester in GP sample was 9.86 mg/L, which presented a sweet, waxy, and floral fragrance.

Acids played a role in enhancing the main aroma and influencing the taste and aftertaste of baijiu. Hexanoic acid (259.91–743.14 mg/L), acetic acid (458.24–607.39 mg/L), and butyric acid (21.23–113.04 mg/L) were the most abundant. Hexanoic acid was a key component of fermented aroma, while butyric acid had a cheesy and sour odor. Alcohols provided aroma and helped enhance the overall flavor profile. 3-Methyl-1-Butanol (18.22–214.02 mg/L) and 1-propanol (20.16–166.10 mg/L) were the most prevalent, consistent with Wang’s findings [34]. Aldehydes, at appropriate levels, can enhance the aroma of baijiu but may impart bitterness if excessive. Furfural (18.44–43.93 mg/L) was relatively abundant across different grades. Notably, GP samples had the highest content of these compounds (over 100 mg/L), while B1 samples had the lowest. Studies have reported that while different brands of NXB share similar volatile compounds, the concentration deviation of volatile compounds contained in them may be helpful for their identification [35]. For instance, Wang et al. [36] used SPME with GC-FID and GC-MS to analyze the volatile compounds in Chinese Daohuaxiang baijiu, quantifying 28 compounds, including ethyl acetate, butanoic acid ethyl ester, pentanoic acid ethyl ester, hexanoic acid ethyl ester, octanoic acid ethyl ester, ethyl isovalerate, ethyl isobutyrate, isoamyl acetate, benzenepropanoic acid ethyl ester, hexanoic acid, 3-methylbutanal, 1-butanol, and 1-hexanol. Their results align with this study.

**Figure 4:**
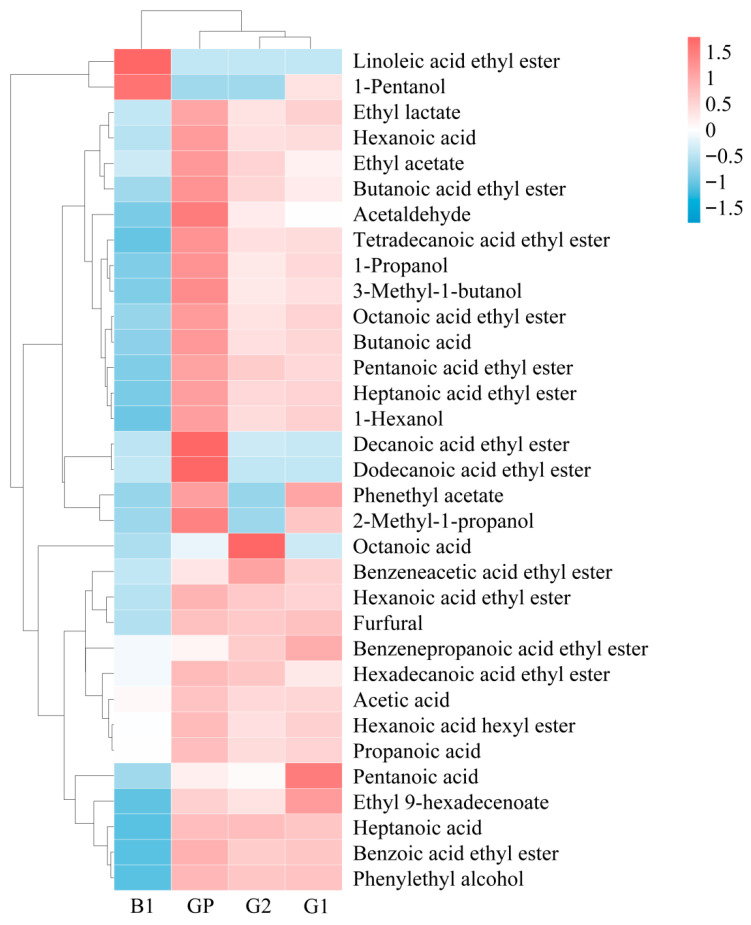
Heatmap analysis of main volatile components in different grades of NXB.

To better understand the content changes in major volatile compounds in NXB, heatmaps were created for different compound categories. As shown in Figure 4, there were certain content differences among different grades of NXB. The clustering results showed that the first-grade baijiu (G1) and the second-grade baijiu (G2) were classified into one category, indicating that the content differences between these two grades were not very significant. The premium-grade baijiu (GP) differed from G2 and G1 in content but had a similar overall profile. The base liquor (B1), with significantly lower flavor compound content than the graded baijius, was classified into a separate category. In G1, compounds such as pentanoic acid, ethyl 9-hexadecenoate, and benzenepropanoic acid ethyl ester had relatively high contents. G2 had higher contents of octanoic acid and benzeneacetic acid ethyl ester. GP generally had higher flavor compound contents than G1 and G2, especially in esters (ethyl acetate, butanoic acid ethyl ester, pentanoic acid ethyl ester, hexanoic acid ethyl ester, ethyl lactate, tetradecanoic acid ethyl ester, hexadecanoic acid ethyl ester, and others), acids (hexanoic acid, butyric acid, acetic acid, and others), alcohols (1-propanol, 2-methyl-1-propanol, 3-methyl-1-butanol, and others), and aldehydes (acetaldehyde). In contrast, B1 had lower contents of most volatile compounds, with only linoleic acid ethyl ester and 1-pentanol being relatively high. Overall, there were significant differences in the contents of various compounds among different grades of NXB. GP samples had the highest compound contents and the greatest difference compared to B1. In the three graded baijius, most esters followed the content order of GP > G1 > G2.

### 3.4. OAV Analysis of Aroma Compounds

The concentration of aromatic compounds in baijiu only reflects their content. A high-concentration compound is not necessarily a major contributor to flavor, and vice versa. Thus, OAV analysis is essential to assess each compound’s flavor importance. Generally, only compounds with OAV ≥ 1 can impact the flavor. The higher the OAV, the greater the compound’s contribution to the baijiu’s overall flavor. To identify the key aroma compounds of NXB in different grades, this study analyzed the OAVs of aroma compounds in various grades. The results were presented in Table 2.

The calculation of OAVs revealed that the number of key aroma compounds with OAV ≥ 1 of NXB in different grades were 21 (G2), 23 (G1), and 26 (GP), markedly higher than in B1 samples (14). This indicated these compounds significantly contribute to the aroma quality of NXB. There were 14 common aroma compounds among all samples, which indicated that these compounds were the key aroma compounds of NXB. Esters account for the majority of these compounds. Compounds like butanoic acid ethyl ester, pentanoic acid ethyl ester, hexanoic acid ethyl ester, and octanoic acid ethyl ester (OAV > 100) were particularly important, especially hexanoic acid ethyl ester (OAV > 10,000). In GP samples, hexanoic acid ethyl ester had an OAV of 24,379.72, confirming its role as the primary aroma compound in NXB, responsible for fermented and fruity aromas. Because of its high content and low threshold [35], it was once again confirmed that the flavor characteristics of NXB were mainly determined by hexanoic acid ethyl ester. These esters were also key aroma compounds in other NXB like Wuliangye, Jiannanchun, and Daohuaxiang [33,36]. Other significant esters included ethyl acetate and benzenepropanoic acid ethyl ester (OAV > 10), which provided fruity, sweet, and floral aromas. Additionally, ethyl lactate, benzeneacetic acid ethyl ester, and hexadecanoic acid ethyl ester, with OAV > 1, were important aroma compounds. These esters were formed during fermentation and aging via esterification of alcohols and fatty acids and typically have fruity, floral, and sweet aromatic attributes [26].

Apart from esters, organic acids like hexanoic acid (103.26–295.23), butyric acid (22.01–117.18), pentanoic acid (4.83–29.68) and acetic acid (2.86–3.80) also had OAV > 1 and were crucial for flavor. Acids, as one of the precursors of ester compounds, played the role of fragrance and taste in baijiu, and were mainly produced by various microorganisms using their acetyltransferase system [23]. Then, under the action of esterase (such as carboxylate hydrolase family, lipase, esterase, and cutinase), ethyl ester was produced from acid substances and ethanol. Hexanoic acid, a major volatile component in NXB, has been confirmed in other studies [33,36]. Acids played a vital role in shaping sensory characteristics. They form the inherent aroma of baijiu with other flavor compounds and have a buffering effect. Therefore, the acidic substances in baijiu had a great relationship with the flavor of baijiu. Proper acid content was essential; too little make the liquor taste dull and short-lasting, while too much leaded to harshness and quality decline. Acids also affected sweetness; overly sour liquors have reduced sweetness and lose the back-sweetness.

Notably, the 14 key aroma compounds shared across all samples were also the key compounds in B1 samples. However, each grade of NXB had its characteristic aroma compounds. In GP samples, decanoic acid ethyl ester (66.33), dodecanoic acid ethyl ester (28.17), tetradecanoic acid ethyl ester (98.02), 3-Methyl-1-butanol (1.19), and acetaldehyde (1204.56) were also important aroma compounds of prime-grade baijiu. Acetaldehyde, with a high OAV, provided a grassy and fruity aroma and was a key aroma compound. Compounds like decanoic acid ethyl ester, benzoic acid ethyl ester, tetradecanoic acid ethyl ester, 1-propanol, 1-hexanol, and acetaldehyde were important for different grades of NXB but absent in the base liquor. Phenethyl acetate, 2-methyl-1-propanol, and furfural mainly contributed to prime-grade and first-grade baijiu. Octanoic acid (5.36) was an important aroma compound in G2 samples.

### 3.5. Key Aroma Compounds of NXB in Different Grades

**Figure 5:**
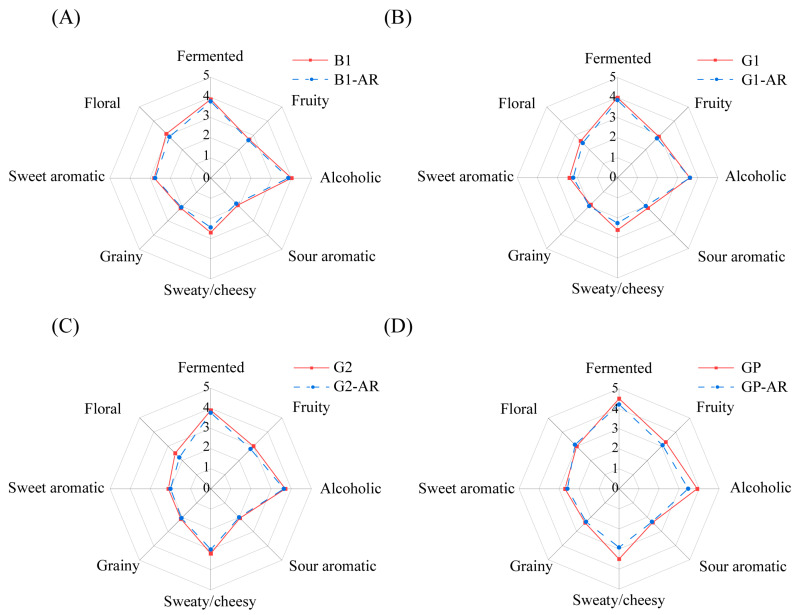
Comparison of sensory contours between different grades of liquor samples and recombinant models (**A**) B1 and its recombinant models B1-AR; (**B**) G2 and its recombination model G2-AR; (**C**) G2 and its recombination model G2-AR; (**D**) GP and its recombinant model GP-AR.

Aroma recombination experiments were conducted to validate all key aroma compounds identified and quantified. The recombination models, created using key aroma compounds from the samples, were compared to actual NXB samples through sensory evaluation. As shown in Figure 5, the radar plot indicated similarity between the recombination models and the baijiu. The models closely matched the baijiu in aroma profile, with differences in eight sensory attributes generally within one point. Sensory assessment suggested that the recombination model provided a more harmonious olfactory experience. Thus, the recombination models successfully replicated the typical aromatic characteristics of NXB in different grades.

To verify the contribution of the aroma compounds in the recombination models to the overall aroma of baijiu samples, omission tests were performed on 30 aroma omission models (each lacking a single compound or a class of compounds), and the results were sensory-evaluated and compared with the complete recombination models. The results of the 30 omission tests were listed in Table 3. The greater the difference between the models before and after omission, the greater the contribution of the omitted compound(s) to the overall aroma profile of NXB in different grades.

Omission model 1 showed that when all esters were omitted, all assessors could distinguish the incomplete model from the complete one across all samples, with significant differences (*p* ≤ 0.001). This indicated that esters, which had fruity and floral aromas, played a decisive role in the overall aroma of NXB. Compared to the complete model, significant differences (*p* ≤ 0.001) occurred when butanoic acid ethyl ester and hexanoic acid ethyl ester were omitted in all samples. Omitting ethyl acetate, pentanoic acid ethyl ester, ethyl lactate, octanoic acid ethyl ester, and hexanoic acid hexyl ester led to differences that increased with the baijiu grade. Omitting decanoic acid ethyl ester only showed differences in the GP sample (0.01 < *p* ≤ 0.05). Omitting benzoic acid ethyl ester caused a difference in G1 (0.01 < *p* ≤ 0.05) and a significant difference in GP (0.001 < *p* ≤ 0.01). Omitting benzeneacetic acid ethyl ester caused differences in B1, G1, and GP, with a significant difference in G2 (0.001 < *p* ≤ 0.01). Omitting phenethyl acetate only affected G1 and GP (0.01 < *p* ≤ 0.05). Omitting dodecanoic acid ethyl ester led to a significant difference in GP (0.001 < *p* ≤ 0.01). Omitting benzenepropanoic acid ethyl ester caused differences in G2 and GP (0.01 < *p* ≤ 0.05) and a significant difference in G1 (0.001 < *p* ≤ 0.01). Omitting tetradecanoic acid ethyl ester caused differences in G2 and G1 (0.01 < *p* ≤ 0.05) and a significant difference in GP (0.001 < *p* ≤ 0.01). Omitting hexadecanoic acid ethyl ester only affected G2 and GP (0.01 < *p* ≤ 0.05).

As shown in omission model 2, the complete omission of organic acids in NXB led to significant differences (*p* ≤ 0.001) between the omission and complete models, as identified by assessors. This highlighted the importance of sour aroma compounds to the overall aroma profile. Compared to the complete model, significant differences (*p* ≤ 0.001) occurred when hexanoic acid was omitted in all samples. Omitting acetic acid and butanoic acid caused differences in B1, G2, and G1 (0.01 < *p* ≤ 0.05), with acetic acid showing a significant difference in GP (0.01 < *p* ≤ 0.05) and butyric acid a highly significant difference (*p* ≤ 0.001). Pentanoic acid omission caused differences in G1 and GP (0.01 < *p* ≤ 0.05), and octanoic acid omission only affected G2 (0.01 < *p* ≤ 0.05). The results indicated that hexanoic acid and butyric acids played a decisive role in the sour aroma of NXB.

Omission model 3 showed that removing all alcohols from the complete recombination model only led to significant differences (0.001 < *p* ≤ 0.01), and assessors failed to identify the omission of all alcohols. Compared to the complete model, significant differences (0.01 < *p* ≤ 0.05) occurred when 1-propanol, 2-methyl-1-propanol, 3-methyl-1-butanol, and 1-hexanol were omitted. In G2, 1-propanol and 2-methyl-1-propanol omission showed differences (0.01 < *p* ≤ 0.05).

Model 4 showed that omitting all aldehydes in B1, G2, and G1 samples caused significant differences (0.001 < *p* ≤ 0.01). In GP samples, this omission led to highly significant differences (*p* ≤ 0.001). Specifically, omitting acetaldehyde resulted in differences in G2 and G1 (0.01 < *p* ≤ 0.05) and a highly significant difference in GP (*p* ≤ 0.001). Furfural omission only affected G1 and GP (0.01 < *p* ≤ 0.05).

**Figure 6:**
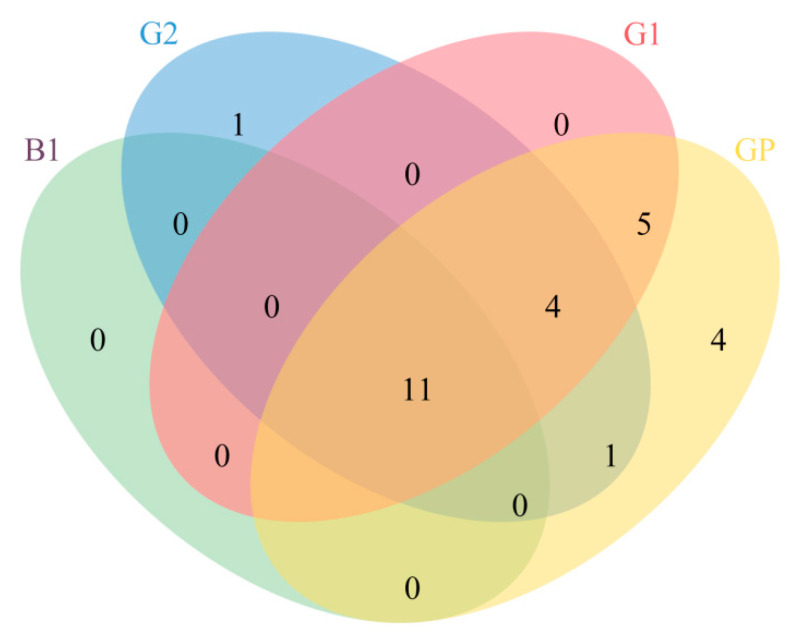
Venn diagram of key aroma compounds in different grades of NXB.

As shown in Figure 6, the key aroma compounds common to all samples were 11 in number. These included ethyl acetate, butanoic acid ethyl ester, pentanoic acid ethyl ester, hexanoic acid ethyl ester, ethyl lactate, octanoic acid ethyl ester, hexanoic acid hexyl ester, benzeneacetic acid ethyl ester, acetic acid, butanoic acid, and hexanoic acid. These compounds have been confirmed as critical aroma compounds of NXB in different brands [15,35,36]. In addition to these shared compounds, different grades of NXB had their own distinctive key aroma compounds. For instance, octanoic acid, which imparted a sweaty/cheesy note, was unique to G2. Both G1 and GP possessed key aroma compounds such as benzoic acid ethyl ester, phenethyl acetate, pentanoic acid, 2-methyl-1-propanol, and furfural. These compounds contributed sweet, floral, sweaty/cheesy, alcoholic, and roast aromas. GP also had its own set of key aroma compounds, including decanoic acid ethyl ester, dodecanoic acid ethyl ester, 3-methyl-1-butanol, 1-hexanol, which provided fruity and alcoholic aromas. Among these, isoamyl acetate, benzeneacetic acid ethyl ester, and octanoic acid have been confirmed as characteristic volatile compounds that can indicate changes in the grade of NXB [37,38]. This implies that although NXB shares fundamental aroma compounds constituting its characteristic profile, distinct quality grades exhibit unique signature aroma components that differentiate their sensory attributes.

## 4. Conclusions

This study delved into the aroma signatures of NXB in different grades. Initially, by comparing sensory evaluation, electronic nose analysis, and three-dimensional fluorescence spectroscopy, it was determined that B1 significantly differed from the graded baijiu. Among the various NXB grades, G1, G2, and GP also exhibited notable differences, with GP boasting the most superior flavor characteristics. Subsequently, a detection of volatile compounds in the samples identified 112 volatile compounds. Among these, hexanoic acid ethyl ester was the most abundant, followed by ethyl acetate and ethyl lactate. Key aroma compounds with OAV ≥ 1 were identified as 21 (G2), 23 (G1), and 26 (GP) in each sample. Aroma recombination models successfully replicated the aroma profiles of NXB in different grades. Omission tests revealed 11 common key aroma compounds critical for the basic flavor of NXB. Additionally, each grade of NXB had distinctive key aroma compounds. G2 had octanoic acid (sweaty/cheesy and sour aroma); G1 had benzoic acid ethyl ester, phenethyl acetate, pentanoic acid, 2-methyl-1-propanol, and furfural (sweet, floral, sweaty/cheesy, alcoholic, and roast aromas). GP had higher aroma compound contents than G1 and G2, with distinctive compounds like decanoic acid ethyl ester, dodecanoic acid ethyl ester, 3-methyl-1-butanol, and 1-hexanol (sweaty/cheesy, fruity, and alcoholic aromas). In summary, the overall aroma characteristics of NXB in different grades were determined by both the types and contents of key aroma compounds. This study systematically reveals consistent differences in key aroma attributes and the composition of main flavor compounds among various grades of NXB by integrating sensory evaluation and instrumental analysis. The established analytical methodology and identified key compounds provide a theoretical foundation for constructing quality grading models based on objective flavor compound data. Furthermore, the multi-technique integrated strategy employed in this research demonstrates significant application potential, showing promise for development into a rapid and accurate quality monitoring tool. This approach offers technical support for gradually transitioning the industry from traditional experience-based grading practices toward a digitalized and standardized quality control system.

In addition, it is worth noting that there are significant differences between NXB in different regions and different manufacturers. It is based on this understanding of the industry’s characteristics that we only selected products from the same region and the same manufacturer but of different grades as the subjects of our study. Our research intention was to focus on the impact of grade differences on flavor characteristics while controlling for variables such as origin, production techniques, and brand as much as possible, in order to explore the relationship between grade and key flavor components under fixed conditions. Due to the inherently complex composition of NXB, fully characterizing all its components remains a daunting challenge that requires extensive further investigation. In future studies, we hope to build upon this foundation by incorporating samples from different regions and brands, while employing additional techniques such as GC-O, sensory taste evaluation, and electronic tongue to more comprehensively reveal the rich profile of NXB.

## Figures and Tables

**Table 1:** Content of main volatile compounds in different grades of NXB.

Compound	Linear Range (mg/L)	Linear Equation	R^2^	Content (mg/L)			
				B1	G2	G1	GP
Ethyl acetate	50.40–5040.30	y = 1.0876x + 8.9314	0.9998	492.16 ± 12.62	901.46 ± 8.46	750.79 ± 9.60	1211.82 ± 10.57
Butanoic acid ethyl ester	20.00–1999.70	y = 1.5369x + 9.4252	0.9998	40.94 ± 0.64	114.67 ± 7.25	99.04 ± 3.42	166.47 ± 2.36
Pentanoic acid ethyl ester	1.01–403.86	y = 1.6892x + 1.4572	0.9999	4.33 ± 0.16	22.68 ± 1.31	20.92 ± 0.71	29.10 ± 1.28
Hexanoic acid ethyl ester	9.94–993.75	y = 1.8108x + 3.0277	0.9998	558.60 ± 3.64	1193.86 ± 4.57	1146.18 ± 1.76	1348.93 ± 9.10
Heptanoic acid ethyl ester	0.22–2228.43	y = 1.9640x + 0.1755	0.9999	0.74 ± 0.12	4.70 ± 0.17	4.82 ± 0.29	6.64 ± 0.57
Ethyl lactate	54.38–5437.80	y = 0.9938x − 1.7341	0.9999	337.98 ± 8.07	603.74 ± 8.10	670.12 ± 7.46	838.89 ± 8.91
Octanoic acid ethyl ester	0.13–131.97	y = 2.0698x − 0.6619	0.9999	1.31 ± 0.10	3.93 ± 0.11	4.33 ± 0.19	5.91 ± 0.37
Hexanoic acid hexyl ester	0.46–92.50	y = 0.8775x + 2.340	0.9956	8.04 ± 0.29	9.91 ± 0.30	10.73 ± 0.18	11.91 ± 0.40
Decanoic acid ethyl ester	0.01–127.73	y = 2.0416x − 3.8535	0.9977	0.05 ± 0.01	3.78 ± 0.21	2.21 ± 0.23	74.44 ± 0.09
Benzoic acid ethyl ester	0.13–133.03	y = 2.0874x − 3.2949	0.9995	ND ^a^	4.77 ± 0.02	4.91 ± 0.02	5.65 ± 0.01
Benzeneacetic acid ethyl ester	0.11–105.47	y = 2.1660x − 5.8542	0.9992	1.82 ± 0.13	4.52 ± 0.06	3.56 ± 0.04	3.14 ± 0.11
Phenethyl acetate	0.12–124.02	y = 2.0417x − 5.0071	0.9993	ND	ND	1.17 ± 0.11	1.21 ± 0.05
Dodecanoic acid ethyl ester	0.09–90.00	y = 1.1319x − 5.4185	0.9984	ND	ND	ND	9.86 ± 0.16
Benzenepropanoic acid ethyl ester	2.62–130.91	y = 2.0345x − 5.6279	0.9992	5.02 ± 0.45	7.23 ± 0.51	8.56 ± 0.23	5.76 ± 0.12
Linoleic acid ethyl ester	0.18–180.00	y = 0.9318x +0.3677	0.9998	20.41 ± 0.76	ND	ND	ND
Tetradecanoic acid ethyl ester	0.08–81.90	y = 0.7825x + 0.4512	0.9984	ND	29.36 ± 0.08	30.13 ± 0.54	49.01 ± 0.51
Hexadecanoic acid ethyl ester	2.57–428.50	y = 0.7891x − 5.6723	0.9985	46.23 ± 0.36	68.34 ± 1.23	56.78 ± 0.92	72.31 ± 0.65
Ethyl 9-hexadecenoate	0.10–97.44	y = 0.3373x − 13.8479	0.9958	ND	12.89 ± 1.14	20.36 ± 0.21	14.84 ± 0.17
Acetic acid	80.77–2019.30	y = 0.7445x − 50.7577	0.9985	458.24 ± 0.03	546.04 ± 1.02	554.63 ± 0.61	607.39 ± 3.01
Propanoic acid	1.99–199.81	y = 1.1913x − 8.5085	0.9984	8.96 ± 0.26	10.99 ± 0.24	11.50 ± 0.30	12.86 ± 0.38
Butanoic acid	8.18–409.16	y = 1.4708x − 18.5989	0.9983	21.23 ± 0.01	74.44 ± 0.01	78.97 ± 0.01	113.04 ± 0.01
Pentanoic acid	0.11–112.68	y = 1.6725x + 7.8123	0.9943	1.88 ± 0.12	5.02 ± 0.68	11.55 ± 0.44	5.64 ± 0.68
Hexanoic acid	7.95–397.50	y = 1.6337x − 19.4822	0.9981	259.91 ± 0.01	516.43 ± 0.01	531.19 ± 0.01	743.14 ± 0.05
Heptanoic acid	0.14–137.70	y = 0.8459x + 6.7832	0.9995	ND	7.02 ± 0.34	6.53 ± 0.09	6.97 ± 0.04
Octanoic acid	0.13–127.40	y = 0.5890x + 8.874	0.9980	ND	14.48 ± 1.11	1.31 ± 0.49	2.80 ± 0.35
1-Propanol	4.04–4041.80	y = 1.7244x + 15.2927	0.9999	20.16 ± 0.80	98.38 ± 3.41	110.65 ± 0.05	166.10 ± 6.02
2-Methyl-1-Propanol	0.39–394.85	y = 2.0471x + 0.6496	0.9999	ND	ND	31.00 ± 0.30	47.96 ± 1.69
1-Pentanol	0.12–119.25	y = 2.1360x + 0.0251	0.9999	3.44 ± 0.23	ND	1.50 ± 0.01	ND
3-Methyl-1-Butanol	8.01–801.36	y = 2.0942x + 1.3986	0.9999	18.22 ± 0.35	117.63 ± 1.47	126.94 ± 3.67	214.02 ± 5.21
1-Hexanol	0.98–97.68	y = 0.4681x − 1.2937	0.9924	1.21 ± 0.02	12.23 ± 0.36	13.32 ± 0.10	17.69 ± 0.14
Phenylethyl Alcohol	0.15–153.70	y = 2.0945x − 8.7875	0.9970	ND	5.18 ± 0.04	5.25 ± 0.08	5.67 ± 0.06
Acetaldehyde	0.38–3861.10	y = 0.4229x + 10.3323	0.9997	ND	281.33 ± 4.90	230.70 ± 2.57	602.28 ± 2.63
Furfural	4.05-–405.45	y = 1.3037x − 1.8545	0.9998	18.44 ± 2.28	41.99 ± 0.59	43.92 ± 1.95	43.93 ± 6.13

^a^ ND meant not detected.

**Table 2:** OAV analysis of aroma compounds.

Compound	Threshold (μg/L)	Aroma Description	OAV			
			B1	G2	G1	GP
Ethyl acetate	32,551.6 [16]	fruity	15.12	27.69	23.06	37.23
Butanoic acid ethyl ester	81.5 [16]	fruity, floral	502.33	1406.99	1215.21	2042.58
Pentanoic acid ethyl ester	26.78 [16]	fruity, floral	161.69	846.9	781.18	1086.63
Hexanoic acid ethyl ester	55.33 [16]	fruity, fermented	10,095.79	21,577.08	20,715.34	24,379.72
Heptanoic acid ethyl ester	13,153.17 [16]	floral, sweet aromatic	<1	<1	<1	<1
Ethyl lactate	128,083.8 [20]	fruity, sweet aromatic	2.64	4.71	5.23	6.55
Octanoic acid ethyl ester	12.87 [16]	fruity, sweet aromatic	101.79	305.36	336.44	459.21
Hexanoic acid hexyl ester	1890 [9]	fruity	4.25	5.24	5.68	6.30
Decanoic acid ethyl ester	1122.3 [16]	fruity, floral	<1	3.37	1.97	66.33
Benzoic acid ethyl ester	1433.65 [16]	honey, floral	ND ^a^	3.33	3.42	3.94
Benzeneacetic acid ethyl ester	407 [13]	honey, floral	4.47	11.11	8.75	7.71
Phenethyl acetate	909 [13]	floral, sweet aromatic	ND	ND	1.29	1.33
Dodecanoic acid ethyl ester	350 [21]	floral, sweet aromatic	ND	ND	ND	28.17
Benzenepropanoic acid ethyl ester	125 [13]	fruity, honey	40.16	57.84	68.48	46.08
Tetradecanoic acid ethyl ester	500 [22]	floral	ND	58.72	60.26	98.02
Hexadecanoic acid ethyl ester	39,299 [18]	fruity, creamy	1.18	1.74	1.44	1.84
Acetic acid	160,000 [13]	sour aromatic	2.86	3.41	3.47	3.8
Propanoic acid	18,100 [9]	sour aromatic	<1	<1	<1	<1
Butanoic acid	964.164 [16]	cheesy, sour aromatic	22.01	77.17	81.86	117.18
Pentanoic acid	389.11 [16]	sweaty, sour aromatic	4.83	12.9	29.68	14.49
Hexanoic acid	2517.16 [16]	sweaty, fermented	103.26	205.16	211.03	295.23
Heptanoic acid	13,821.32 [16]	sweaty, sour aromatic	ND	<1	<1	<1
Octanoic acid	2701.23 [16]	cheesy, sour aromatic	ND	5.36	<1	1.04
1-Propanol	53,952.63 [16]	alcoholic	<1	1.82	2.05	3.08
2-Methyl-1-propanol	28,300 [13]	alcoholic, malt	ND	ND	1.1	1.69
1-Pentanol	6400 [19]	alcoholic	<1	ND	<1	ND
3-Methyl-1-butanol	179,190.83 [16]	alcoholic, malt	<1	<1	<1	1.19
1-Hexanol	2500 [17]	alcoholic	<1	4.89	5.33	7.08
Phenylethyl alcohol	28,900 [13]	floral	ND	<1	<1	<1
Acetaldehyde	500 [1]	grass, fresh	ND	562.66	461.4	1204.56
Furfural	44,029.73 [16]	roast	<1	<1	1	1

^a^ ND meant not detected.

**Table 3:** Omission experiments.

Model	Compounds Removed from the Recombinant Model	Significant Difference ^a^			
		B1	G2	G1	GP
1	All ester compounds	***	***	***	***
1ND1	Ethyl acetate	*	**	*	**
1-2	Butanoic acid ethyl ester	***	***	***	***
1-3	Pentanoic acid ethyl ester	*	**	**	***
1-4	Hexanoic acid ethyl ester	***	***	***	***
1-5	Ethyl lactate	*	*	**	***
1-6	Octanoic acid ethyl ester	*	**	**	***
1-7	Hexanoic acid hexyl ester	*	*	*	**
1-8	Decanoic acid ethyl ester	ND ^b^	-	-	*
1-9	Benzoic acid ethyl ester	ND	-	*	**
1-10	Benzeneacetic acid ethyl ester	*	**	*	*
1-11	Phenethyl acetate	ND	ND	*	*
1-12	Dodecanoic acid ethyl ester	ND	ND	ND	**
1-13	Benzenepropanoic acid ethyl ester	-	*	**	*
1-14	Tetradecanoic acid ethyl ester	ND	*	*	**
1-15	Hexadecanoic acid ethyl ester	-	*	-	*
2	All acid compounds	***	***	***	***
2-1	Acetic acid	*	*	*	**
2-2	Butanoic acid	*	*	*	***
2-3	Pentanoic acid	-	-	*	*
2-4	Hexanoic acid	***	***	***	***
2-5	Octanoic acid	ND	*	ND	-
3	All alcohol compounds	**	**	**	**
3-1	1-Propanol	ND	*	*	*
3-2	2-Methyl-1-propanol	ND	ND	*	*
3-3	3-Methyl-1-butanol	ND	ND	ND	*
3-4	1-Hexanol	ND	-	-	*
4	All aldehyde compounds	**	**	**	***
4-1	Acetaldehyde	ND	*	*	***
4-2	Furfural	ND	ND	*	*

^a^ Significance: *, significant (0.01 < *p* ≤ 0.05); **, highly significant (0.001 < *p* ≤ 0.01); ***, very highly significant (*p* ≤ 0.001); -, not significant. ^b^ ND meant not detected.

## Data Availability

The original contributions presented in this study are included in the article/Supplementary Materials. Further inquiries can be directed to the corresponding authors.

## Supplementary Materials

The following supporting information can be downloaded at: https://www.mdpi.com/article/10.3390/foods14213781/s1, Figure S1: Proof of ethical approval.
