# Supplementary material for: Comprehensive Investigation of Grade-Specific Aroma Signatures in Nongxiangxing Baijiu Using Flavoromics Approaches
Source: Foods. 2025 Nov 4;14(21):3781. doi: 10.3390/foods14213781 (PMC12610442; doi:10.3390/foods14213781)
Supplement: Supplementary file 1 [file foods-14-03781-s001.zip › foods-3889405-supplementary.pdf]

Supplementary Material

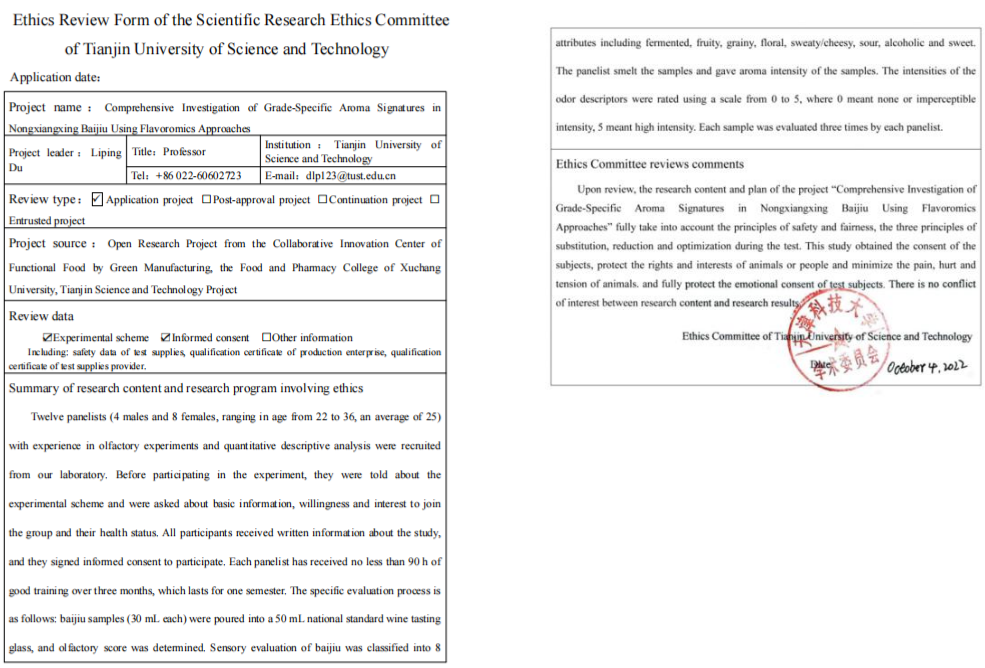

Ethics Review Form of the Scientific Research Ethics Committee of Tianjin University of Science and Technology

Application date:

Project name: Comprehensive Investigation of Grade-Specific Aroma Signatures in Nongxiangxing Baijiu Using Flavoromics Approaches		
Project leader: Liping Du	Title: Professor	Institution: Tianjin University of Science and Technology
	Tel: +86 022-60602723	E-mail: dlp123@tust.edu.cn
Review type: ☑ Application project ☐ Post-approval project ☐ Continuation project ☐ Entrusted project		
Project source: Open Research Project from the Collaborative Innovation Center of Functional Food by Green Manufacturing, the Food and Pharmacy College of Xuchang University, Tianjin Science and Technology Project		
Review data<br>☑ Experimental scheme ☑ Informed consent ☐ Other information<br>Including: safety data of test supplies, qualification certificate of production enterprise, qualification certificate of test supplies provider.		
Summary of research content and research program involving ethics<br>Twelve panelists (4 males and 8 females, ranging in age from 22 to 36, an average of 25) with experience in olfactory experiments and quantitative descriptive analysis were recruited from our laboratory. Before participating in the experiment, they were told about the experimental scheme and were asked about basic information, willingness and interest to join the group and their health status. All participants received written information about the study, and they signed informed consent to participate. Each panelist has received no less than 90 h of good training over three months, which lasts for one semester. The specific evaluation process is as follows: baijiu samples (30 mL each) were poured into a 50 mL national standard wine tasting glass, and olfactory score was determined. Sensory evaluation of baijiu was classified into 8		

attributes including fermented, fruity, grainy, floral, sweaty/cheesy, sour, alcoholic and sweet. The panelist smelt the samples and gave aroma intensity of the samples. The intensities of the odor descriptors were rated using a scale from 0 to 5, where 0 meant none or imperceptible intensity, 5 meant high intensity. Each sample was evaluated three times by each panelist.
Ethics Committee reviews comments<br>Upon review, the research content and plan of the project "Comprehensive Investigation of Grade-Specific Aroma Signatures in Nongxiangxing Baijiu Using Flavoromics Approaches" fully take into account the principles of safety and fairness, the three principles of substitution, reduction and optimization during the test. This study obtained the consent of the subjects, protect the rights and interests of animals or people and minimize the pain, hurt and tension of animals. and fully protect the emotional consent of test subjects. There is no conflict of interest between research content and research results.<br>Ethics Committee of Tianjin University of Science and Technology<br>Date: October 4, 2022

Figure S1 Proof of ethical approval
